# Availability of Personal Protective Equipment (PPE) Among US and Pakistani Doctors in COVID-19 Pandemic

**DOI:** 10.7759/cureus.8550

**Published:** 2020-06-10

**Authors:** Jawad Ahmed, Farheen Malik, Taha Bin Arif, Zainab Majid, Muhammad A Chaudhary, Junaid Ahmad, Mehreen Malik, Taj M Khan, Muhammad Khalid

**Affiliations:** 1 Internal Medicine, Dow University of Health Sciences, Karachi, PAK; 2 Family Medicine, WellSpan Good Samaritan Hospital, Lebanon, USA; 3 Center for Surgery and Public Health, Harvard Medical School/Harvard T. H. Chan School of Public Health, Boston, USA; 4 Internal Medicine, Liaquat University of Medical and Health Sciences, Jamshoro, PAK; 5 Anesthesiology, Aga Khan University, Karachi, PAK; 6 Pediatrics, Baylor College of Medicine/Texas Children's Hospital, Houston, USA; 7 Cardiology, Kansas City University of Medicine and Biosciences, Joplin, USA; 8 Cardiology, Ascension Via Christi Hospital, Pittsburg, USA

**Keywords:** covid-19, personal protective equipment, ppe, healthcare workers, stop transmission, doctors, physicians, infection, sars-cov-2, pandemic

## Abstract

Background

The coronavirus disease (COVID-19) pandemic has put an excessive strain on healthcare systems across the globe, causing a shortage of personal protective equipment (PPE). PPE is a precious commodity for health personnel to protect them against infections. We investigated the availability of PPE among doctors in the United States (US) and Pakistan.

Methods

A cross-sectional study, including doctors from the US and Pakistan, was carried out from April 8 to May 5, 2020. An online self-administered questionnaire was distributed to doctors working in hospitals in the US and Pakistan after a small pilot study. All analysis was done using Statistical Package for Social Science (SPSS) version 23.0 (IBM Corp., Armonk, NY).

Results

After informed consent, 574 doctors (60.6% from Pakistan and 39.4% from the US) were included in the analysis. The majority of the participants were females (53.3%), and the mean age of the participants was 35.3 ± 10.3 years. Most doctors (47.7%) were from medicine and allied fields. Among the participants, 87.6% of doctors from the US reported having access to masks/N95 respirators, 79.6% to gloves, 77.9% to face-shields or goggles, and 50.4% to full-suit/gown. Whereas, doctors in Pakistan reported to have poor availability of PPE with only 37.4% having access to masks/N95 respirator, 34.5% to gloves, 13.8% to face-shields or goggles, and 12.9% to full-suit/gown. The reuse of PPE was reported by 80.5% and 60.3% physicians from the US and Pakistan, respectively. More doctors from Pakistan (50.6%) reported that they had been forced to work without PPE compared to doctors in the US (7.1%).

Conclusion

There is a lack of different forms of PPE in the US and Pakistan. Doctors from both countries reported that they had been forced to work without PPE. Compared to the US, more doctors from Pakistan reported having faced discrimination in receiving PPE.

## Introduction

The novel coronavirus, initially originating from the Hubei province of China, has spread to nearly every continent, overwhelming and straining even the most sophisticated healthcare systems [1]. The Severe Acute Respiratory Syndrome Coronavirus 2 (SARS-CoV-2) is transmitted through inhalation or contact with infectious droplets. It may be asymptomatic early on in the course or present with mild respiratory symptoms, headache, fever, fatigue, nausea, vomiting, in addition to some reports describing hematological and cardiac involvement [2]. Healthcare workers (HCWs) or individuals who tend to coronavirus disease (COVID-19) patients are at highest risk of contracting the infection.

The prevention of the spread of infection to and from medical personnel solely lies in the effective use of personal protective equipment (PPE), including gloves, face masks, air-purifying respirators, goggles, face shields, respirators, and gowns. The rampant nature of COVID-19 has created a shortage of PPE in high demand areas. The abrupt increase in the demand for PPE has to be met with an accelerated manufacturing and supply of PPE. Many healthcare systems are failing to provide PPE due to financial or time constraints. There have been multiple reports of HCWs protesting about the lack of appropriate PPE, and instances of doctors and other healthcare staff being forced into working without this precious commodity [3,4].

The healthcare system of the United States (US) is known all over the world for its innovative and highly specialized patient care. The US spends a significant amount (17.1%) of the country's Gross Domestic Product (GDP) on health care, which is far more on health care as a percentage of its economy than any other developed nation [5]. Nonetheless, the US has the highest number of cases (1,432,265 cases and 87,180 deaths; May 18, 2020) of COVID-19, and despite being the pinnacle of modern medicine, the healthcare system is strained and stretched to its very limits [6].

As expected, the situation in developing countries, with weaker healthcare infrastructure, is even direr. The COVID-19 pandemic was confirmed to have reached Pakistan on February 26, 2020 [6]. In Pakistan, 42,125 cases have been reported with 903 deaths till May 18, 2020 [6]. Consequently, the already struggling health care system of Pakistan is not equipped for large pouring in of potentially infectious patients seeking testing and care [7]. Resources are stretched thin, and the number of HCWs being infected is rising every day. In this context, we sought to examine the availability of PPE in Pakistan (a resource-constraint country) and the US (resource-rich country) as well as draw a comparison between the two in terms of availability, discrimination in distribution and perceived reasons for the shortage of PPE.

## Materials and methods

Study design and duration

A cross-sectional study was carried out among doctors in the US and Pakistan using convenience sampling. The study duration was from April 8 to May 5, 2020.

Study population and inclusion criteria

A self-administered questionnaire was made using Google Forms and was distributed to doctors in the US and Pakistan via emails and social media platforms. The bias of receiving irrelevant (non-doctors) responses was reduced by posting the questionnaire on doctor/physician-only groups. The credibility of social media groups was ensured that they only admit licensed doctors after confirming their registration numbers and affiliations. The inclusion criteria consisted of three points, (1) a practicing doctor, (2) work in a hospital, and (3) currently working in Pakistan or the US. Responses of doctors working in private clinics were excluded from the study. Explanation of the study's aim, as well as the informed consent form was present at the start of the questionnaire, permitting us to collect the data.

Study tool

A structured questionnaire consisting of four major parts was designed by authors (see appendices section). The initial draft of the questionnaire was sent to multiple senior doctors for evaluation, and all appropriate suggestions were incorporated in the questionnaire. The first part consisted of a brief explanation of the study, informed consent statement, and demographic variables such as country name, age, gender, the specialty of work, and hospital type (private or public). The second part consisted of questions to assess the availability of different forms of PPE, including N95 respirator, masks, gloves, eye protection (goggles or face shield), and full-suit/gown. The third part consisted of questions to assess any discrimination in PPE distribution, perceived reasons for its shortage, the extent of reuse of PPE, and to identify if doctors had been forced to work without PPE. The last part consisted of questions about the likelihood of quitting the job if adequate PPE was not provided and feelings of doctors working in the pandemic situation. In total, the questionnaire consisted of 33 items.

A small pilot study was carried out among 10 doctors from each country (Pakistan and the US) to ensure that no ambiguity exists in the questionnaire. Recent contact with COVID-19 patients was defined as a contact within the last three days of filling the questionnaire.

Statistical analysis

All the data were entered and analyzed through Statistical Package for the Social Sciences software (SPSS version 23.0; IBM Corporation, Armonk, NY, US). Results were drawn through descriptive statistics, and means with standard deviation were presented for continuous variables such as age and amount of money spent on purchasing PPE. Categorical variables were reported as frequencies with percentages. Chi-squared and Independent sample T-tests were used to find statistical significance, and a p-value of <0.05 was considered significant for all analyses.

## Results

A total of 574 doctors (268; 46.7% males) from both countries (60.6%; n = 348 from Pakistan and 39.4%; n = 226 from the US) were included in the analysis. The mean age of participants was 35.3 ± 10.3 years, and most of the respondents (47.7%; n = 274) were from medicine and allied fields. The demographics of the participants are noted in Table 1.

**Table 1:** Demographics of the study US: United States

Variable	Options	Total (n = 574)	Pakistan (n = 348)	United States (n = 226)	P-value and comments
Gender	Females	306 (53.3)	196 (56.3)	110 (48.7)	P = 0.204
	Males	268 (46.7)	152 (43.7)	116 (51.3)	
Age groups (years)	Mean age (years)	35.3 ± 10.3	31.8 ± 9.4	40.8 ± 9.2	P < 0.001
	≤30	256 (44.6)	224 (64.4)	32 (14.2)	The majority of doctors from Pakistan and the US were ≤30 and 31-40 years, respectively.
	31-40	160 (27.9)	76 (21.8)	84 (37.2)	
	41-50	92 (16)	22 (6.3)	70 (31.0)	
	51-60	52 (9.1)	16 (4.6)	36 (15.9)	
	>61	14 (2.4)	10 (2.9)	4 (1.8)	
Hospital type	Public	404 (70.4)	298 (85.6)	106 (46.9)	-
	Private	170 (29.6)	50 (14.4)	120 (53.1)	
Department of work	Medicine and allied	274 (47.7)	146 (42.0)	128 (56.6)	Most doctors were from medicine and allied departments, but a significant difference was found between the departments in both countries (p = 0.016)
	Pediatrics	98 (17.1)	72 (20.7)	26 (11.5)	
	Surgery and allied	114 (19.9)	78 (22.4)	36 (15.9)	
	Emergency medicine	70 (12.2)	38 (10.9)	32 (14.2)	
	Pathology and lab	6 (1.0)	2 (0.6)	4 (1.8)	
	Miscellaneous (radiology and anesthesia)	12 (2.1)	12 (3.4)	0 (0.0)	

Availability of PPE in the US and Pakistan

PPE availability was reported significantly more (p < 0.001) among doctors in the US than doctors in Pakistan. In the US, 53.1% (n = 120) doctors reported having access to all forms of PPEs (including N95 respirators/masks, gloves, gowns/full-suits, and face-shields or goggles) in their hospitals, whereas only 12.6% (n = 44) of doctors in Pakistan reported having this privilege.

Among the participants, 87.6% (n = 198) of doctors from the US reported having access to masks/N95 respirator, 79.6% (n = 180) to gloves, 77.9% (n = 176) to face-shields or goggles, and 50.4% (n = 114) to full-suit/gown. In contrast, doctors in Pakistan reported to have poor availability of PPE with only 37.4% (n = 130) having access to masks/N95 respirator, 34.5% (n = 120) to gloves, 13.8% (n = 48) to face-shields or goggles, and 12.9% (n = 44) to full-suit/gown. More than half (58.4%; n = 132) of N95 respirator users in the US reported to have size-fitted masks, whereas only a quarter (25.9%; n = 90) of participants from Pakistan reported size-fitting for their N95 respirator in Pakistan.

Reuse of PPE

**Figure 1:**
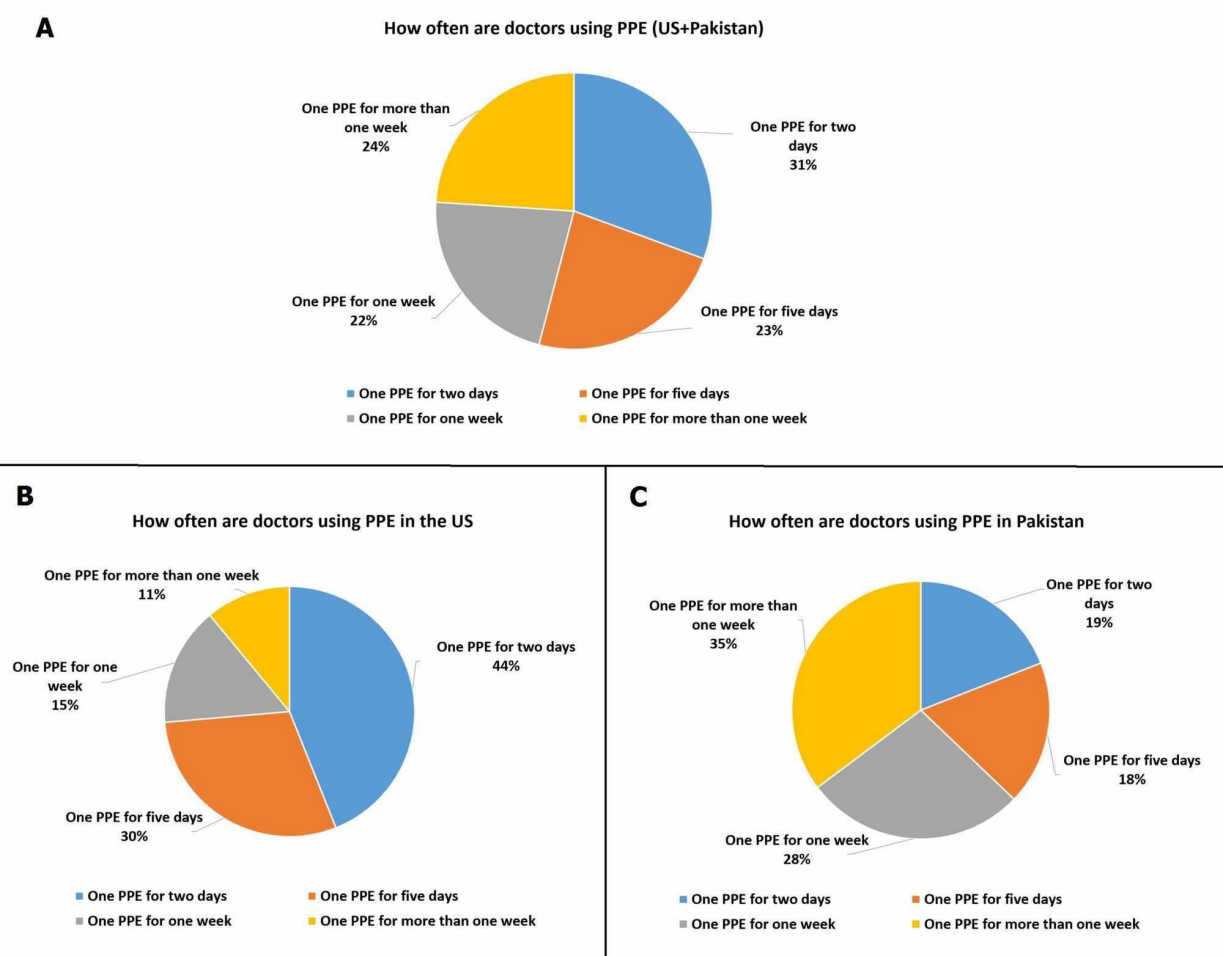
Responses of doctors regarding the reuse of PPE: (A) Total, (B) the US, and (C) Pakistan PPE: Personal protective equipment; US: United States

In our analysis, 80.5% (n = 182) and 60.3% (n = 210) doctors from the US and Pakistan reported reusing PPE, respectively. The details of the frequency of reusing PPE for the US and Pakistan are graphically presented in Figure 1. Fewer doctors in the US responded to have bought PPE themselves compared to Pakistan (49.4%; n = 172 vs. 35.4%; n = 80). Likewise, the average expenditure of buying PPE reported by Pakistani doctors was higher than doctors working in the US (mean expenditure $19.34 and $14.33, respectively).

Discrimination in receiving PPE

A total of 280 (48.8%) doctors from both countries responded to have faced discrimination in receiving PPE. According to the responses we received, significantly fewer doctors in the US faced discrimination in getting PPE (8%; n = 18) when compared to Pakistan (75.3%; n = 262). Only doctors from Pakistan (3.4%; n = 12) reported gender and racial discriminations in the distribution of PPE. Other causes of discrimination of PPE are noted in Table 2.

**Table 2:** Responses of doctors from the US and Pakistan to different questions *Others include religious beliefs, marital status, personal issues with distributing authority, and first-come-first-serve basis. PPE: Personal protective equipment; HCWs: Healthcare workers; COVID-19: Coronavirus disease; US: United States. Chi-squared test and Independent sample T-test were used to find statistical significance between the variables.

Questions	Variables/Options	Total (n = 574)	Pakistan (n = 348)	United States (n = 226)	P-value and comments
Number of doctors who faced any discrimination in receiving PPE		280 (48.8)	262 (75.3)	18 (8.0)	Pakistani doctors reported to have faced significantly higher discrimination in receiving PPE compared to the US (p < 0.001).
Basis of discrimination in PPE distribution	Race/gender	12 (2.1)	12 (3.4)	0 (0.0)	
	Lack of seniority in position e.g. a junior/new doctor	98 (17.1)	92 (26.4)	6 (2.7)	
	Nepotism	52 (9.1)	50 (14.4)	2 (0.9)	
	Provided only to HCWs in COVID-19 wards	94 (16.4)	84 (24.1)	10 (4.4)	
	Others*	24 (4.2)	24 (6.9)	0 (0.0)	
Number of doctors who reported reusing PPE more than once		392 (68.3)	210 (60.3)	182 (80.5)	A significant difference was noted in the reuse of PPE with countries (p < 0.001).
How often are doctors using PPE	One PPE for two days	120 (20.9)	40 (11.5)	80 (35.4)	
	One PPE for five days	92 (16.0)	38 (10.9)	54 (23.9)	
	One PPE for one week	86 (15.0)	58 (16.7)	28 (12.4)	
	One PPE for more than one week	94 (16.4)	74 (21.3)	20 (8.8)	
Perceived reason for lack of PPE	Shortage of supply	392 (68.3)	192 (55.2)	200 (88.5)	Shortage of supply was perceived as the main reason for the lack of PPE in our study.
	Inadequate/poor distribution management	236 (41.1)	146 (42.0)	90 (39.8)	
	Rigged system/political issues	164 (28.6)	152 (43.7)	12 (5.3)	
	Increased prices	152 (26.5)	94 (27.0)	58 (25.7)	
How likely are doctors to quit their job if they do not receive proper PPE in the future?	Never quit	72 (12.5)	32 (9.2)	40 (17.7)	Our results show a significant difference (p = 0.024) in the response of both countries. Pakistan doctors reported a higher likelihood of quitting.
	Unlikely to quit	148 (25.8)	76 (21.8)	72 (31.9)	
	Unsure about quitting	182 (31.7)	118 (33.9)	64 (28.3)	
	Likely to quit	140 (24.4)	98 (28.2)	42 (18.6)	
	Definitely quit	32 (5.6)	24 (6.9)	8 (3.5)	
Feelings of doctors working in pandemic	Scared	288 (50.2)	174 (50.0)	114 (50.4)	No significant difference was noted between feelings variable with country (p = 0.173) in our research.
	Unbothered	34 (5.9)	14 (4.0)	20 (8.8)	
	Feeling purposeful and proud of themselves	162 (28.2)	110 (31.6)	52 (23.0)	
	Unsure how they feel in such a situation	90 (15.7)	50 (14.4)	40 (17.7)	

In the absence of PPE, significantly higher (p < 0.001) number of doctors in Pakistan (81.0%; n = 282) reported to have kept working in contrast to doctors form the US (14.2%; n = 32). Moreover, a significantly higher (p < 0.001) number of doctors from Pakistan (49.4%; n = 172) reported that they had been "bullied into working" without PPE as opposed to the US doctors (7.1%; n = 16).

Perceived reasons for the unavailability of PPE

**Figure 2:**
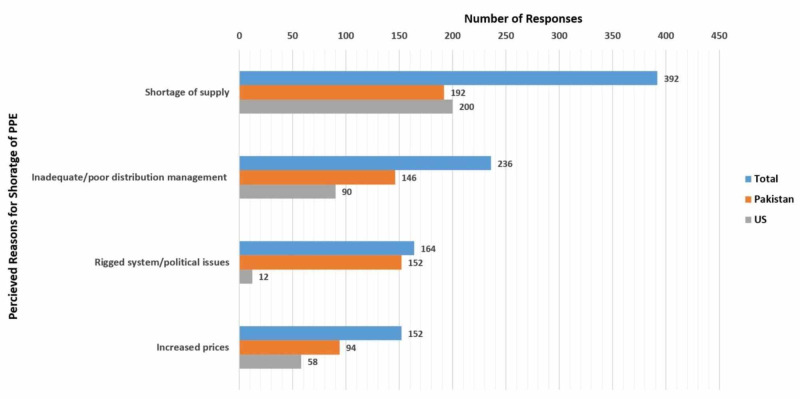
Perceived reasons for the lack of PPEs as reported by doctors PPE: Personal protective equipment; US: United States

Shortage of supply (68.3%; n = 392) and inadequate/poor distribution management (41.1%; n = 236) were reported as the most common reasons for the lack of PPE (Table 2). Other reasons reported by doctors from the US and Pakistan are given in Figure 2.

Feelings of doctors working in pandemic

A total of 140 (24.4%) doctors, with 98 (28.2%) from Pakistan and 42 (18.6%) from the US reported that they would "likely quit" their job and 32 (5.6%) doctors (the US = 8; Pakistan = 24) proclaimed that they would "definitely quit" if they do not receive proper PPE in the future (Table 2).

**Figure 3:**
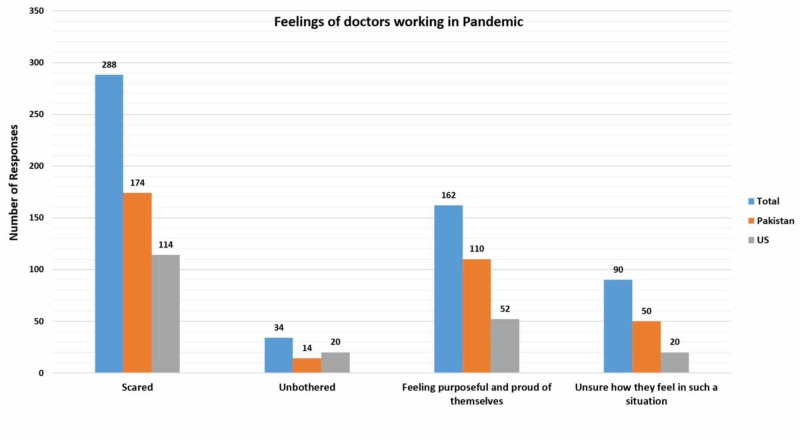
Feelings of doctors working in pandemic situation US: United States

Almost three-fourth (73%; n = 254) doctors from Pakistan and 58.4% (n = 132) from the US reported that HCWs in their hospitals had been infected by COVID-19. Half of the respondents from the US (50.4%; n = 114) and Pakistan (50.0%; n = 174) expressed that they felt scared working in a pandemic situation (Table 2). Almost one-third (31.6%; n = 110) doctors from Pakistan and one-fourth (23%; n = 52) doctors from the US reported that they felt purposeful and proud of themselves for working in the pandemic (Figure 3).

The summarized results of the study and responses of doctors from both countries are presented in Table 2 and Table 3.

**Table 3:** Responses of doctors related to remaining questions regarding the lack of personal protective equipment and their explanations PPE: Personal protective equipment; HCW: Healthcare worker; WHO: World Health Organization; COVID-19: Coronavirus disease; US: United States. Chi-squared test and Independent sample T-test were used to find statistical significance between the variables.

Questions	Pakistan (n = 348)		United States (n = 226)		P-value	Comments
	Yes (%)	No (%)	Yes (%)	No (%)		
Are you currently working on the frontline with COVID-19 patients?	222 (63.8)	126 (36.2)	176 (77.9)	50 (22.1)	0.011	-
Have you recently (within the past three days) been in contact with positive/suspected COVID-19 patients?	332 (95.4)	16 (4.6)	210 (92.9)	16 (7.1)	0.371	No significant difference.
Do you have access to all forms of PPEs (masks/N95 respirators, gloves, full-suit, hair caps, and eye protection) in your hospital?	Access to all = 44 (12.6)		Access to all = 120 (53.1)		<0.001	In our study, doctors in the US reported to have better access to PPEs compared to Pakistan, where 14.4% of doctors had no access to any PPE.
	Access to some = 254 (73)		Access to some = 106 (46.9)			
	Access to none = 50 (14.4)		Access to none = 0 (0.0)			
Are you getting proper PPE according to WHO guidelines?	62 (17.8)	286 (82.2)	156 (69.0)	70 (31.0)	<0.001	Based on the responses we received, the availability of PPE is more in the US as compared to Pakistan.
Do you have mask/N95 respirators available for use when needed?	130 (37.4)	218 (62.6)	198 (87.6)	28 (12.4)	<0.001	
If you use N95 respirator, were you size fitted for it?	90 (25.9)	196 (56.3)	132 (58.4)	90 (39.8)	<0.001	
Do you have gloves for use when needed?	120 (34.5)	228 (65.5)	180 (79.6)	46 (20.4)	<0.001	
Do you have a full-suit available for use when needed?	44 (12.9)	304 (87.4)	114 (50.4)	112(49.6)	<0.001	
Do you have face shields or goggles for use when needed?	48 (13.8)	300 (86.2)	176 (77.9)	50 (22.1)	<0.001	
Do you have access to hand sanitizer/disinfectants readily?	224 (64.4)	124 (35.6)	222 (98.2)	4 (1.8)	<0.001	
Is there discrimination in the distribution of PPE among your department or hospital staff?	262 (75.3)	86 (24.7)	18 (8.0)	208 (92.0)	<0.001	They are discussed in Table 2 with reported reasons.
Have you bought any PPE from your pocket?	172 (49.4)	176 (50.6)	80 (35.4)	146 (64.6)	0.019	In our study, more Pakistani doctors reported to have bought PPE compared to doctors in the US.
In some instances in the past, if there were no PPE, did you continue working without any protective equipment?	282 (81.0)	66 (19.0)	32 (14.2)	194 (85.8)	<0.001	In our study, a significantly higher number of doctors from Pakistan reported that they kept working without PPE.
Does the lack of PPE make you feel like quitting your job?	Yes = 160 (46.0)		Yes = 72 (31.9)		0.006	Based on responses, a significantly higher number of doctors in Pakistan reported that lack of PPE makes them feel like quitting their job.
	No = 72 (20.7)		No = 84 (37.2)			
	Maybe = 116 (33.3)		Maybe = 70 (31.0)			
Have you ever been bullied into working without PPE?	172 (49.4)	176 (50.6)	16 (7.1)	210 (92.9)	<0.001	Doctors in Pakistan reported a significantly higher incidence of bullying compared to the US.
Has any HCW in your hospital infected with COVID-19?	254 (73.0)	94 (27.0)	132 (58.4)	94 (41.6)	0.10	No significant difference.
Does the lack of PPE make you feel scared for your loved ones as you might carry the infection to your home?	344 (98.9)	4 (1.1)	220 (97.3)	6 (2.7)	0.341	No significant difference.

## Discussion

One of the most substantial strategies to protect both patients and HCWs from transmittable pathogens is the adequate use of PPE. In our study, the availability of PPE was reported to be better among the US doctors as compared to Pakistani doctors. More doctors in Pakistan faced discrimination in receiving PPE, and the reuse of PPE was reported by the doctors from both countries.

According to the World Health Organization (WHO), the essential supplies of PPE include gowns, gloves, masks or respirators, goggles, face shields, head cover, and rubber boots. Since COVID-19 is primarily transmitted by contact or droplet and its definite cure has not been discovered yet, the only significant and emotive subject for the HCWs is PPE. The types of protection required to combat the specific mode of transmission include (1) gloves and aprons as contact precautions, (2) gloves, aprons, fluid-resistant surgical masks with or without eye protection (goggles or a visor) for droplet transmissions, and (3) gloves, fluid repellant long-sleeved gowns, eye protection, and filtering facepiece 2/3 (FFP2/3) mask or N95 respirator during aerosol-generating procedures [8].

Our study population comprised of doctors from two countries having a vastly different landscape of healthcare and helped us in comparing the difficulties faced by both countries in the face of a pandemic.

Availability of PPE

A comparison of PPE availability in the US and Pakistan with reports from the UK is shown in Table 4. Our results are consistent with reports from the UK that some protective equipment such as gowns/full-suits and eye protection are scarce [4]. Almost three-quarters of the doctors from Pakistan reported that they were not size fitted for their N95 respirator. These results are alarming as the improper fitting of the N95 respirator reduces its efficacy and can make doctors susceptible to infection [9].

**Table 4:** Comparison of availability of different forms of PPE in the US, Pakistan, and the UK * The percentage is for FFP3 (filtering facepiece 3) mask. PPE: Personal protective equipment; US: United States; UK: United Kingdom.

Country	Face mask/N95 respirator	Gloves	Eye protection (face shields or goggles)	Full-suit or gown
United States	87.6%	79.6%	77.9%	50.4%
Pakistan	37.4%	34.5%	13.8%	12.9%
United Kingdom [4]	72%*	77%	43%	49%

In our study, doctors from the US reported to have comparatively better access to PPEs, however, they too, are struggling to maintain adequate PPE supply in light of the overwhelming influx of cases, and are not out of danger to get infected. For the worst-hit cities like New York and San Francisco, donations were called for from the locals, to ease the state of desperation [1]. The strategic national stockpile (SNS), which was responsible for making PPEs available during epidemics of Ebola virus and H1N1 influenza, is currently making an effort to balance between a quick distribution and restocking [1]. Part of the problem could be attributed to the unpreparedness of the authorities despite multiple warnings of a possible influenza pandemic in the near future [10]. Furthermore, the US had decreased its production of masks, gowns, and gloves and hugely relied on imports from countries like China [10].

Reuse of PPE

In our study, more than one-third of the doctors from Pakistan (35%) reported reusing one PPE for more than one week, while most of the doctors in the US (44%) reported reusing one PPE for two days. In times of extreme shortages and the rapidly increasing cases, the Health and Safety Executive (HSE) recently issued guidance that recommended reusing PPEs, which followed a skeptical response by the HCWs of England [11]. The guidance stated reusing water-resistant equipment and promoted the use of sealable bags for storage, whereas washable gowns or similar long-sleeved articles of clothing were advised as replacements for medical gowns [11]. In literature, several methods of disinfection have been described, which include the use of hydrogen peroxide vapors, ultraviolet (UV) radiation, moist heat, dry heat, and ozone gas, with hydrogen peroxide vapors being the most widely suggested technique [12]. Reuse of FFP after appropriate measures has been considered a suitable alternative; however, it is uncertain to state the same for surgical masks [12]. Although our study reported that a large majority of physicians from the US and Pakistan reusing their pieces of equipment, the exact method of decontamination being administered is beyond the scope of this study and needs further evaluation.

Discrimination in receiving PPE

The dire shortage of protective gear does not seem to be the only concern of HCWs worldwide as the biased distribution and supply further add to the trouble. Analysis of our data showed that doctors from Pakistan faced greater discrimination in receiving PPE, with one of the main reasons being lack of seniority in position, which is surprising since a large workforce in tertiary care hospitals comprises of junior training doctors.

Our study showed that 49.4% and 7.1% of doctors from Pakistan and the US, respectively, were forced to work without PPE. When compared to the reports from the UK, where 56% of doctors felt pressurized to work despite inadequate PPE, our figures are lower, but they cannot be ignored [4]. News of doctor being arrested for demanding PPE in Pakistan has also been reported [13].

Reasons for the unavailability of PPE

Several factors have contributed to the shortage of PPEs on a global scale. One reason is the psychological 'fear of uncertainty' among masses, giving rise to panic buying and hoarding of masks and gloves along with other essential products. In the current era of globalization, the supply of any product is dependent on its demand; however, with the advent of this sudden calamity, the demand has escalated multiple folds in a short duration, leaving the suppliers struggling to keep up. Disruption in the supply and demand graph has resulted in a higher equilibrium price, and certain opportunists are trading the life-saving essentials at staggering rates. Another major aspect contributing to the crisis is the travel/export restrictions halting China's trade, which produces and supplies nearly 50% of the worldwide face masks along with other types of safety equipment [14].

The shortage of supplies on a global scale has not only rendered several nations to improvise and innovate but also highlighted the significance of national self-dependency. WHO, at the beginning of March 2020, advised the relevant industries and governments to ramp up the manufacturing of PPEs by 40% to curb the ever-increasing demand [15]. Regardless of the efforts of several non-governmental organizations (NGOs) in Pakistan to distribute PPEs among doctors and donation of supplies from China as a gesture of goodwill, the level of protection of Pakistan's HCWs is nowhere near satisfactory. In the US, however, the role of NGOs in contributing to PPE supply or healthcare system is limited, the reason being the notable difference in the socioeconomic status and spendings of both countries on their respective health budgets. Even before the pandemic, the government-run tertiary-care hospitals of Pakistan highly relied on the interventions from NGOs and overseas donors, with the dependency increasing now more than ever.

Feelings of doctors

The majority of doctors in our study reported being scared of the current situation and feared that they might transmit the infection to their loved ones. The feeling is synchronous among doctors globally, particularly emergency room physicians, working in direct contact with the infected patients, and performing the intubation [16]. The fear of infecting loved ones was responded positively by the vast majority of both Pakistani and the US doctors, with 98.9% and 97.3% responses, respectively. The figure is comparatively greater than that found in a survey performed by Royal College of Physicians (RCP), stating that 61% of respondents worrying about spreading the disease to family members [17].

Almost 50% of participants of our study, from the US and Pakistan, described feeling scared while working in a global pandemic. The study from RCP reported similar data, with 48% of their respondents feeling concerned about working in the current situation [17]. The fear among HCWs is real and justified as the infectivity and mortality due to COVID-19 is increasing every day. Similarly, a study on the effect of the SARS outbreak (2002-2009) on HCWs described a significant sense of threat to life, vulnerability, along with the somatic and cognitive impact [18].

In our study, about 160 (46%) Pakistani participants felt like quitting their job due to lack of PPE, which was higher than the response from the US doctors, 72 (31.9%) of which responded affirmatively. However, this figure is not in line with a study from Greece published during the A/H1N1 influenza pandemic in 2009, where only 4.3% of HCWs reported opting for leave to avoid contracting the virus [19]. Even a recent article from the same country (Greece) reported the willingness of HCWs to work during the current pandemic to be unaffected [20]. The readiness to work, in turn, may be dependent on the socioeconomic status of a state, the trust of its service workers on government, and the severity of the situation. Other factors may include but are not limited to gender, childcare responsibilities, personal safety, and protective measures [21].

In our study, 73% of doctors from Pakistan and 58.4% from the US reported that HCWs in their hospitals had been infected with COVID-19. In China, an estimated 3,300 HCWs were infected, and 22 expired due to "insufficient protective equipment" [22]. These statistics are alarming, for the war against COVID-19 is yet to be over. As the number of infected/dead HCW rises, the anxiety and reluctance of other HCWs to work will also increase [23].

Recommendations and limitations

The general public should be made aware of the fact that PPE is crucial for HCWs on the frontline and N95 respirators, full-suits/gowns or eye protection are not required for daily life uses. It is suggested to introduce a proper surveillance system for the distribution of PPE among doctors in a healthcare setting. Social media spreading unauthentic information and promoting black marketing to escalate prices should be banned. The shortage of PPE can cripple the healthcare system. As more healthcare personnel will get infected with COVID-19, the workforce fighting against pandemic will decrease. A recent study conducted in a resource-limited setting found that ward assistants have not been adequately educated about hygiene protocols [24]. Educating ward assistants is equally important regarding the proper use of hand disinfectants and PPE, so they do not act as a vector for transmitting infections to healthy patients in this time of COVID-19 pandemic.

Alternative methods for the conservation of these limited and indispensable commodities need to be employed. At present, some healthcare systems are disinfecting or reprocessing PPEs using appropriate techniques reported by WHO or using telemedicine tools for performing medical exams as a form of electronic-PPE [25,26]. Furthermore, recommendations of reprocessing N95 respirators using hydrogen peroxide vapors or UV light have surfaced. Although resourceful, these methods lack standardized protocols, and the efficacy of the disinfection process is still uncertain [25].

Our study is limited by its online-survey nature, small sample size, and reporting bias, as some responses may be driven by personal emotions of doctors.

## Conclusions

To conclude, there is a shortage of PPE in hospitals of the US and Pakistan due to COVID-19 and doctors are feeling scared working without adequate protection in the pandemic situation. Some doctors even reported that they are likely to quit their job if they do not receive proper PPE in the future. Adequate PPE is crucial in the battle against COVID-19, and radical steps need to be taken by hospital administrations and governments to make PPE more accessible to doctors and other HCWs. There is a need to educate the general population regarding PPE usage and make them realize that doctors and other HCWs are the ones that need them the most. Further research is required on this topic that includes other healthcare personnel, and a need to study the opinion of the general population regarding PPE usage is suggested.

## Appendices

Questionnaire

Availability of personal protective equipment (PPE) is a pressing issue for doctors working in COVID-19 pandemic. The study aims to assess the availability of PPE to all doctors currently working. The data will be kept confidential and no names and institution details are required. If you meet the following criteria, only then please fill the form (1) are a practicing doctor, (2) work in a hospital (if you work in a private clinic you are ineligible to fill the questionnaire), and (3) working in Pakistan or United States.

By filling the questionnaire you agree to be part of the study and agree to provide information to the best of your knowledge. The study data will be used for publication in any journal.

Thank you for your time and taking part in the study.

Questions

1.       Do you give consent to be a part of this study?

a.      Yes

b.      No 

2.      Gender

a.      Male

b.      Female

3.      Age (years) ______________

4.      Country:  

a.      Pakistan

b.      United States

5.      Type of hospital setting you are working in?  

a.      Public

b.      Private

6.      Department in hospital e.g., cardiology, internal medicine, obstetrics, emergency medicine, etc.___________

7.      Department of work?

a.      Medicine and allied

b.      Pediatrics

c.      Surgery and allied

d.      Emergency Medicine

e.      Pathology and Lab

f.       Miscellaneous such as radiology or anesthesia

8.      Are you currently working on frontline with COVID-19 patients?

a.      Yes

b.      No

9.      Have you recently been in contact with positive/suspected COVID-19 patient?

a.       Yes

b.      No

10.      Are you getting proper PPE based on your workplace according to WHO guidelines? (PPE includes gloves, medical masks, goggles or a face shield, and gowns, as well as for specific procedures, respirators i.e. N95 or FFP2 standard or equivalent and aprons: Source WHO). 

a.      Yes

b.      No

11.   What are the options available to you? (Surgical Mask, gown, head cap, full suit, face shield or goggles, N95 respirators)

a.      All of above

b.      Some of above

c.      None

12.   What are your preventive measures? (Surgical Mask, gown, head cap, full suit, face shield or goggles, N95 respirators)

a.      All of above

b.      Some of above

c.      None

13.   Do you have mask/ N95 respirators available for use when needed?

a.      Yes

b.      No

14.   If you use N95 respirator, were you size fitted for it?

a.      Yes

b.      No

15.   Do you have gloves available for use when needed?

a.      Yes

b.      No

16.   Do you have full suit/gown available for use when needed?

a.      Yes

b.      No

17.   Do you have face shields or goggles for use when needed?

a.      Yes

b.      No

18.   Do you have access to hand sanitizer/ disinfectants readily?

a.      Yes

b.      No

19.   Is there a discrimination in distribution of PPE among your department or hospital?

a.      Yes

b.      No

20.   If yes then based on what?

a.      Race/ gender

b.      Lack of seniority in position e.g.  a junior/new doctor

c.      Nepotism

d.      Provided to healthcare workers directly in COVID 19 wards only

e.      Other

21.   If you chose other, please describe the reason________________________

22.   Are you reusing your PPE more than once?

a.      Yes

b.      No

c.      Not applicable

23.   If yes, how often?

a.      One PPE for 2 days

b.      One PPE for 5 days

c.      One PPE for one week

d.      One PPE for more than 1 week

24.   What do you think is the reason for lack of PPE?

a.      Shortage of supply

b.      Inadequate/poor distribution management

c.      Rigged system/political issues

d.      Increased prices

25.   Have you bought any PPE from your pocket?

a.      Yes

b.      No

26.   If yes, then please write the estimated cost in PKR or USD

________________

27.   At some instance in past, if there were no PPE, did you continue working without any protective equipment?

a.      Yes

b.      No

28.   Does lack of PPE make you feel like quitting your job?

a.      Yes

b.      No

c.      Maybe

29.   With increasing incidence of COVID-19, If you don’t get proper PPE in future how likely are you to quit your job?

a.      Never quit

b.      Unlikely to quit

c.      Unsure about it

d.      Likely to quit

e.      Definitely quit

30.   Have you ever been bullied into working without PPE?

a.      Yes

b.      No

31.   Has any healthcare worker in your hospital setting infected with COVID-19?

a.      Yes

b.      No

32.   How do you feel working in the pandemic situation?

a.      Scared

b.      Unbothered

c.      Feeling purposeful and proud of yourself

d.      Unsure how you feel

33.   Does lack of PPE make you feel scared for your loved ones as you might carry the infection to your home?

a.      Yes

b.      No

Thank you for taking part in the study. Your response has been recorded.
